# Proteomics analysis reveals elevated RAB21 in serum-derived extracellular vesicles from patients with follicular thyroid carcinoma

**DOI:** 10.3892/ol.2025.15190

**Published:** 2025-07-16

**Authors:** Kyojiro Kawakami, Naoki Edo, Koji Morita, Toshio Ishikawa, Hiroyuki Onose, Tatsuya Fukumori, Hiroki Tsumoto, Keitaro Umezawa, Masafumi Ito, Yuri Miura

**Affiliations:** 1 Proteome Research, Tokyo Metropolitan Institute for Geriatrics and Gerontology, Tokyo 173-0015, Japan; 2 Department of Internal Medicine, Teikyo University School of Medicine, Tokyo 173-8605, Japan; 3 Department of Internal Medicine, Kanaji Thyroid Hospital, Tokyo 114-0015, Japan; 4 Department of Surgery, Kanaji Thyroid Hospital, Tokyo 114-0015, Japan; 5 Biological Process of Aging, Tokyo Metropolitan Institute for Geriatrics and Gerontology, Tokyo 173-0015, Japan

**Keywords:** EV, FTC, proteomics analysis, biomarker, serum

## Abstract

Follicular thyroid carcinoma (FTC) is a common thyroid malignancy that poses diagnostic challenges because of its cytological similarity to benign follicular thyroid adenoma (FTA). The present study aimed to identify characteristic protein signatures in serum-derived extracellular vesicles (EVs) of FTC and FTA for potential diagnostic and therapeutic applications. Serum EVs from patients with FTC and FTA were purified using the phosphatidylserine affinity method. Proteomics analysis via nano liquid chromatography-tandem mass spectrometry identified 18 significantly differentially expressed proteins between the two patient groups. RAB21, a small GTPase involved in cellular trafficking, was markedly elevated in serum EVs from patients with FTC. Furthermore, cell invasion and migration assays of a human FTC cell line revealed that RAB21 knockdown reduced cell migratory ability, suggesting its role in the malignant phenotype of FTC. The present findings indicated that RAB21 in serum EVs may be a candidate biomarker able to distinguish FTC from FTA, and that RAB21 could be a potential therapeutic target for FTC.

## Introduction

Papillary thyroid carcinoma (PTC) and follicular thyroid carcinoma (FTC) are major components of differentiated primary thyroid carcinoma. PTC is the most common type, followed by FTC, with the latter accounting for approximately 10–15% of all thyroid malignancies (1). Although both originate from thyroid cells, they have rather different clinical behaviors. PTC frequently invades adjacent organs, such as the recurrent laryngeal nerve, esophagus, and trachea, but this is rare in FTC. Regarding metastases and recurrences, PTC frequently metastasizes to regional lymph nodes, whereas distant metastasis/recurrence is predominant for FTC (2). When determining diagnoses, it is important to differentiate both malignancies from benign types; namely, between PTC and adenomatous nodule (AN) and between FTC and follicular thyroid adenoma (FTA). Cytological examination, with relatively low invasiveness, can distinguish PTC from AN. However, preoperative differentiation between FTC and FTA has remained difficult, because FTC and FTA have overlapping cytomorphological features and the diagnosis of FTC requires histopathological proof of tumor capsule invasion and/or vascular invasion. Although FTC, like PTC, generally has a favorable prognosis if appropriately managed, its prognosis can be limited in patients with certain clinicopathological features. Therefore, diagnostic surgery is recommended for patients presenting with suspicious follicular nodules, with the aim of evaluating the presence of capsular or vascular invasion; however, the malignancy rate is quite low, leading to a large proportion of suspicious follicular nodules being pathologically confirmed as benign tumors after surgery (3,4). Based on these limitations, there is an urgent need to develop a preoperative noninvasive approach to discriminate between FTC and FTA because this would enable the accurate diagnosis of thyroid follicular tumors in clinical practice.

Extracellular vesicles (EVs) are membrane particles released by cells. Although various classification systems exist for EVs, they are commonly categorized based on their size, such as small EVs (50–200 nm) and large EVs (200–1000 nm), or by their biogenesis pathways, such as exosomes and microvesicles (5). Exosomes, a type of small EV, are formed through the endosomal pathway, a cellular membrane trafficking route. They originate from early endosomes, progress to multivesicular bodies, and are released into the extracellular space. A notable characteristic of exosomes is that they contain proteins, nucleic acids, and lipids from the cells from which they derive (6).

EVs are present not only in the immediate vicinity of the cells that release them, but also in bodily fluids, including the blood. This suggests that analyzing EVs released by diseased tissues and circulating in the bloodstream could provide insights into pathological conditions. Consequently, EVs are considered a promising tool for liquid biopsy and are expected to facilitate disease diagnosis (7,8).

In light of their potential, our study implemented an unbiased exploratory approach to compare the protein profiles of serum EVs between FTC and FTA patients. Through the comprehensive proteomic screening of EVs using nano liquid chromatography-tandem mass spectrometry (nanoLC-MS/MS), we aimed to identify differentially expressed proteins that could distinguish between these conditions. Rather than focusing on predetermined targets, we sought to discover novel biomarkers and potential therapeutic targets for FTC, addressing the critical need for the improved preoperative diagnosis of thyroid follicular tumors.

## Materials and methods

### Patients

The protocol of this study was approved by the Medical Review Boards of Teikyo University (approval no. 14-019-6), Tokyo Metropolitan Institute for Geriatrics and Gerontology (approval no. 6179), and Kanaji Thyroid Hospital (approval no. 9). All methods were carried out in accordance with the relevant guidelines and regulations (Declaration of Helsinki). Serum from patients with FTC (10 patients), FTA (14 patients), and follicular tumor of uncertain malignant potential (FT-UMP) (1 patient) was collected between May 2015 and January 2018, and the serum samples were stored at −80°C until use. Patient demographics are summarized in Table SI. Pathological diagnoses were made according to the WHO classification (5th edition) (9).

### Purification of EVs

EVs were purified from human serum by the phosphatidylserine affinity method (10,11). Briefly, the extracellular domain of mouse Tim4 and the Fc region of human IgG1 (FUJIFILM Wako Pure Chemical, Osaka, Japan) were biotinylated and conjugated to streptavidin magnetic beads (Thermo Fisher Scientific, Waltham, MA, USA). Serum (500 µl) was centrifuged at 10,000 × g for 30 min and then filtered through a 0.22-µm filter. The filtered sample was diluted twofold with Tris-buffered saline (TBS) and incubated for 1 h at room temperature (RT) with Tim4-conjugated beads in the presence of 2 mM CaCl_2_. After washing with 0.01% Tween-TBS containing 2 mM CaCl_2_, the captured EVs were eluted with 2 mM EDTA in phosphate-buffered saline (PBS). Purified EVs were stored at −80°C until later use.

### Measurement of protein concentration

The protein concentrations of purified EVs were quantified using the CBQCA protein quantitation kit (Thermo Fisher Scientific) according to the manufacturer's protocol. The fluorescence intensity was measured using an EnVision plate reader (PerkinElmer, Waltham, MA, USA).

### Measurement of EV size

The size of the purified EVs was measured using the qNano particle analyzer (Izon Science, Christchurch, New Zealand). Purified EVs were diluted in 0.01% Tween-PBS buffer and measured using an NP100 nanopore. Calibration was performed using CPC100 as a standard control. Data were analyzed using Izon control software (version 3.4.2.51).

### Western blot analysis

Purified EVs were mixed with sample buffer (62.5 mM Tris-HCl pH 6.8, 2% SDS, 10% glycerol, 0.01% bromophenol blue, and 5% 2-mercaptoethanol) and 130 ng of protein was loaded for SDS-polyacrylamide gel electrophoresis. Equal protein loading was used as there is no established loading control for EVs, particularly for human serum-derived EVs where the amount of purified EVs varies significantly between individuals even from the same volume. The separated proteins were then transferred to PVDF membranes (Millipore, Burlington, MA, USA). After blocking with 1% skim milk, the membranes were probed with a primary antibody, followed by a horseradish peroxidase (HRP)-linked secondary antibody. After washing, bound proteins were visualized using the ImmunoStar LD (FUJIFILM Wako Pure Chemical) as an HRP substrate and the FUSION Chemiluminescence Imaging System (Vilber, Marne-la-Vallée, France) as a detection instrument. The antibodies used in this study were as follows: anti-Alix (ab186429; Abcam, Cambridge, UK), anti-flotillin-2 (610383; BD Transduction Laboratories, Franklin Lakes, NJ, USA), anti-CD9 (13174; Cell Signaling Technology, Danvers, MA, USA), anti-RAB21 (NBP2-93771; Novus Biologicals, Centennial, CO, USA), and anti-GAPDH (2118; Cell Signaling Technology), along with secondary anti-mouse IgG (715-035-151) and anti-rabbit IgG (711-035-152) antibodies (Jackson ImmunoResearch, West Grove, PA, USA). The signal intensities of the obtained data were measured using ImageJ software (version 1.54d) (https://imagej.net/software/imagej/). Uncropped western blot images are provided in Fig. S1.

### Proteomic analysis

Protein digestion with trypsin was performed prior to proteomic analysis. EVs were dissolved in 0.1% Rapigest SF (Waters, Milford, MA, USA), reduced with 10 mM dithiothreitol in 50 mM triethylammonium bicarbonate (TEAB) at RT for 30 min, and then alkylated with 55 mM iodoacetamide in 50 mM TEAB at RT for 30 min in the dark. The samples were digested with trypsin (Promega, Madison, WI, USA) in 50 mM TEAB at 37°C for 16 h. The cleaved peptides were desalted using a GL-Tip SDB column (GL Sciences, Tokyo, Japan) and evaporated in a SpeedVac vacuum concentrator (Thermo Fisher Scientific). The peptides were reconstituted in 0.1% formic acid (FA) and subjected to nanoLC-MS/MS analysis.

Measurements were performed by nanoLC-MS/MS using an Ultimate 3000 RSLCnano system (Thermo Fisher Scientific) coupled to a Q Exactive hybrid quadrupole-Orbitrap mass spectrometer with a nanoESI source (Thermo Fisher Scientific), as previously described (12). The nanoLC system was equipped with a trap column (C18 PepMap 100, 0.3×5 mm, 5 µm particle size; Thermo Fisher Scientific) and an analytical column (NTCC-360/75-3-125, 75 µm × 12 cm, 3 µm particle size, C18; Nikkyo Technos, Tokyo, Japan). Peptides were separated over a period of 90 min using a gradient of water containing 0.1% FA (mobile phase A) and acetonitrile containing 0.1% FA (mobile phase B) at a flow rate of 300 nl/min. The elution gradient was set as follows: 0–3 min, 2% B; 3–93 min, 2–40% B; 93–95 min, 40–95% B; 95–105 min, 95% B; 105–107 min, 95–2% B; and 107–120 min, 2% B. The mass spectrometer was operated in data-dependent acquisition mode. All data were analyzed for protein identification and label-free quantification using Proteome Discoverer 2.4 software (Thermo Fisher Scientific). The analytical parameters used for the database search were as follows: search engine, Sequest HT; protein database, Swiss-prot (Homo Sapiens); enzyme name, trypsin (full); dynamic modification, oxidation (methionine); static modification, carbamidomethyl (cysteine); precursor mass tolerance: 10 ppm, fragment mass tolerance: 0.02 Da, missed cleavage: 2, and false discovery rate (FDR) <0.05. The protein abundances were normalized to the median of all proteins detected in each sample and compared between FTC and FTA using NormalyzerDE (https://normalyzerde.immunoprot.lth.se/) (13).

### Gene expression analysis of FTC using a public database

To estimate the expressions of genes in thyroid cancer tissues, we used the Gene Expression Omnibus (GEO) database (https://www.ncbi.nlm.nih.gov/gds/). We analyzed the GSE15045 dataset, which comprises gene expression data from 4 FTA patients and 8 FTC patients, using the GEO2R web tool (https://www.ncbi.nlm.nih.gov/geo/geo2r/). This analysis allowed us to compare the gene expression profiles of FTA and FTC tissues.

### Cell culture

The human FTC cell line, FTC-133, was obtained from the European Collection of Authenticated Cell Cultures (UK Health Security Agency, Porton Down, UK). FTC-133 cells were cultured in DMEM/Ham's F12 medium (FUJIFILM Wako Pure Chemical) containing 10% fetal bovine serum (FBS) (Thermo Fisher Scientific), 100 units/ml penicillin, and 100 µg/ml streptomycin (FUJIFILM Wako Pure Chemical).

### Small interfering RNA transfection

Dicer-substrate short interfering RNAs (DsiRNAs) for the human RAB21 gene (109142326) and negative control DsiRNA (51-01-14-03) were purchased from Integrated DNA Technologies (Coralville, IA, USA). The sequences of DsiRNAs are shown in Table SII. DsiRNAs were transfected into FTC-133 cells at a concentration of 2 nM using the Lipofectamine RNAiMax transfection reagent (13778150; Thermo Fisher Scientific), in accordance with the manufacturer's instructions. After 6 h of incubation, the medium was changed to DMEM/Ham's F-12 medium supplemented with 10% FBS. After a 2-day culture, the transfected cells were used for western blot analysis, and cell invasion and migration assays.

### Cell invasion assay

DsiRNA-transfected cells were seeded at 2×10^4^ cells onto a Matrigel invasion chamber (354480; Corning, Corning, NY, USA) and incubated at 37°C under a 5% CO_2_ atmosphere. After 24 h, the cells were fixed with 100% methanol and stained with crystal violet, and then, the number of invaded cells was determined. The invasion rate was represented as the ratio of the number of invaded cells for RAB21-knockdown cells compared with that for control cells.

### Cell migration assay

Transfected cells were cultured in a six-well plate until they reached confluence. Cell layers were wounded using a 1 ml pipette tip and cultured for 30 h. The wound area was analyzed based on photographs taken at 0 and 30 h using ImageJ software, and the cell migration rate was calculated from the ratio of each void area at 30 h to that at 0 h, as follows:

Migration rate (%)=[1-(void area at 30 h/void area at 0 h)]x100.

### Statistical analysis

The statistical significance of differences was determined using limma (14) for proteomic analysis. Unpaired Student's t-test was used for analysis of RAB21 in serum-derived EVs from FTC and FTA patients. For analysis of RAB21 mRNA expression from the GEO database, Welch's t-test was performed. For cell invasion and migration assays, data were analyzed using Mann-Whitney U test. P<0.05 was considered to indicate a statistically significant difference.

## Results

### Confirmation of purified EVs

**Figure 1.**
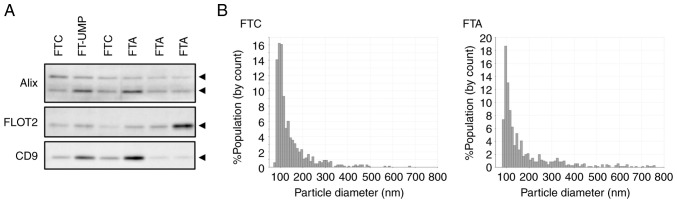
Features of EV fractions purified using the phosphatidylserine affinity purification method. (A) Western blot images of EV protein markers. Uncropped images of blots are shown in Fig. S1A. (B) Representative images of the particle size distribution from FTC and FTA patients. The particle size was measured using a qNano particle size analyzer. FLOT2, flotillin-2; EV, extracellular vesicle; FTA, follicular thyroid adenoma; FTC, follicular thyroid carcinoma; FT-UMP, follicular tumor of uncertain malignant potential.

Serum EVs from patients were purified using the Tim4 molecule, which has affinity for phosphatidylserine. The purified serum EVs were confirmed by detecting EV marker proteins and measuring their particle size characteristics. The analysis of serum EVs purified from patients revealed the presence of the EV markers Alix, Flotillin-2, and CD9, which are commonly found in small EVs (Figs. 1A and S1A). Furthermore, the particle sizes were mainly distributed around 100 nm, which corresponds to the size of small EVs, and there were no substantial differences in this variable among the patients (Fig. 1B).

### Proteomics of serum EVs purified from FTA and FTC patients

**Figure 2.**
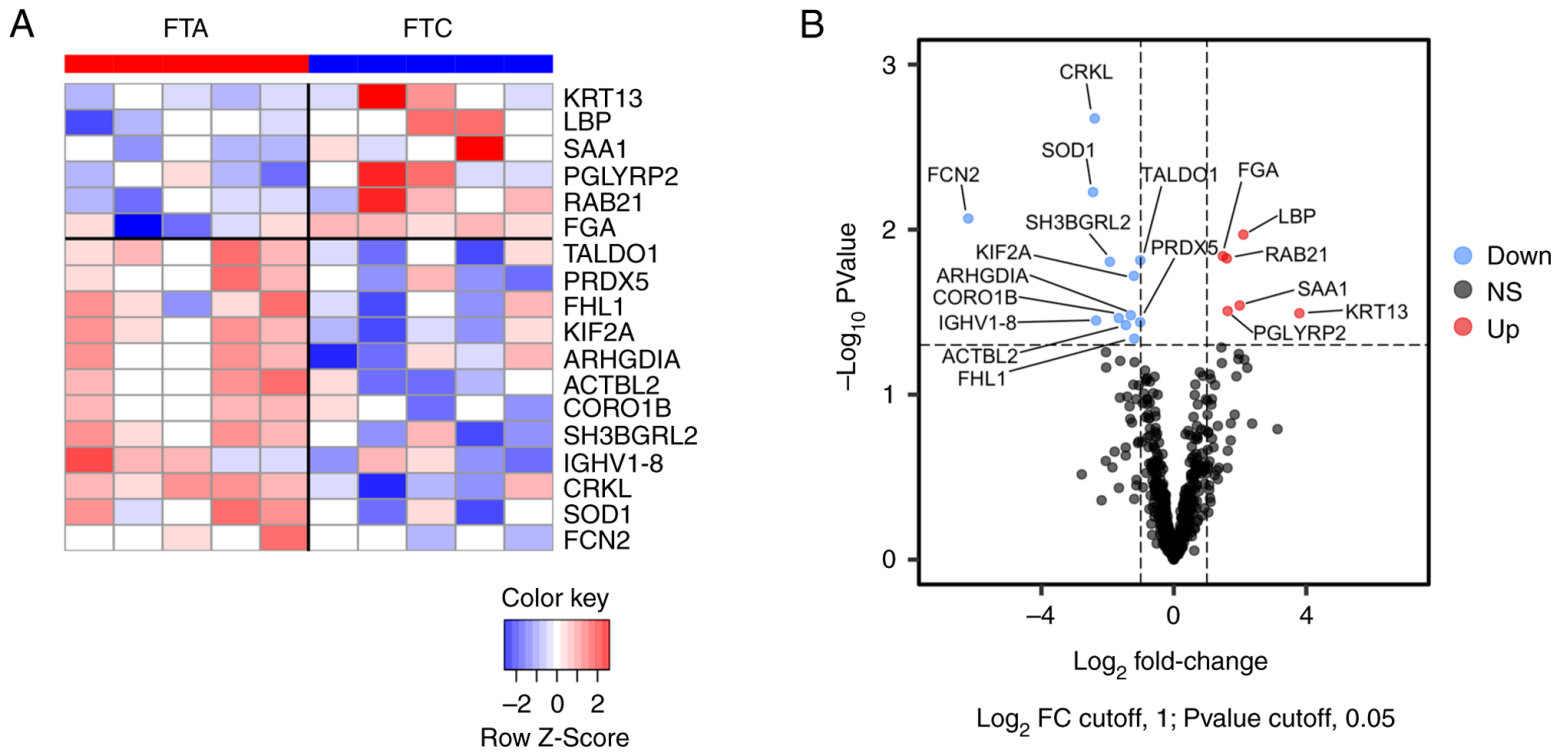
Heatmap and volcano plot of proteomic analysis of EVs from FTC and FTA patients. (A) Heatmap of differentially expressed proteins in EVs derived from FTC patients. (B) Volcano plot of all proteins detected in serum EVs from patients. Raw data of fold changes and P-values are summarized in Table SIII. Down, protein downregulated in FTC; NS, not significant; Up, protein upregulated in FTC; EVs, extracellular vesicles; FTA, follicular thyroid adenoma; FTC, follicular thyroid carcinoma.

Proteomic analysis was performed on the EVs purified from the serum of FTC and FTA patients using an LC-MS/MS instrument to identify differentially expressed proteins. The results identified 639 proteins (Table SIII). Upon comparing the normalized protein abundances between FTC and FTA EVs, we found that 6 proteins had more than a 2-fold increase and 12 proteins had less than 0.5-fold expression in EVs from FTC patients (Fig. 2 and Table I).

Several proteins related to cancer development and progression were identified in the list of abundant proteins in FTC, including Ras-related protein Rab-21 (RAB21) (15), Keratin 13 (KRT13) (16), and Serum amyloid A1 (SAA1) (17). In contrast, the deficient proteins in FTC included antioxidative proteins such as Peroxiredoxin 5 (PRDX5), Superoxide dismutase 1 (SOD1), and Transaldolase (TALDO1). Furthermore, Four and a half LIM domains protein 1 (FHL1), which is a tumor suppressor gene in thyroid cancer (18,19), was downregulated in FTC.

### Validation of upregulated RAB21 in EVs purified from the serum of FTC patients

**Figure 3.**
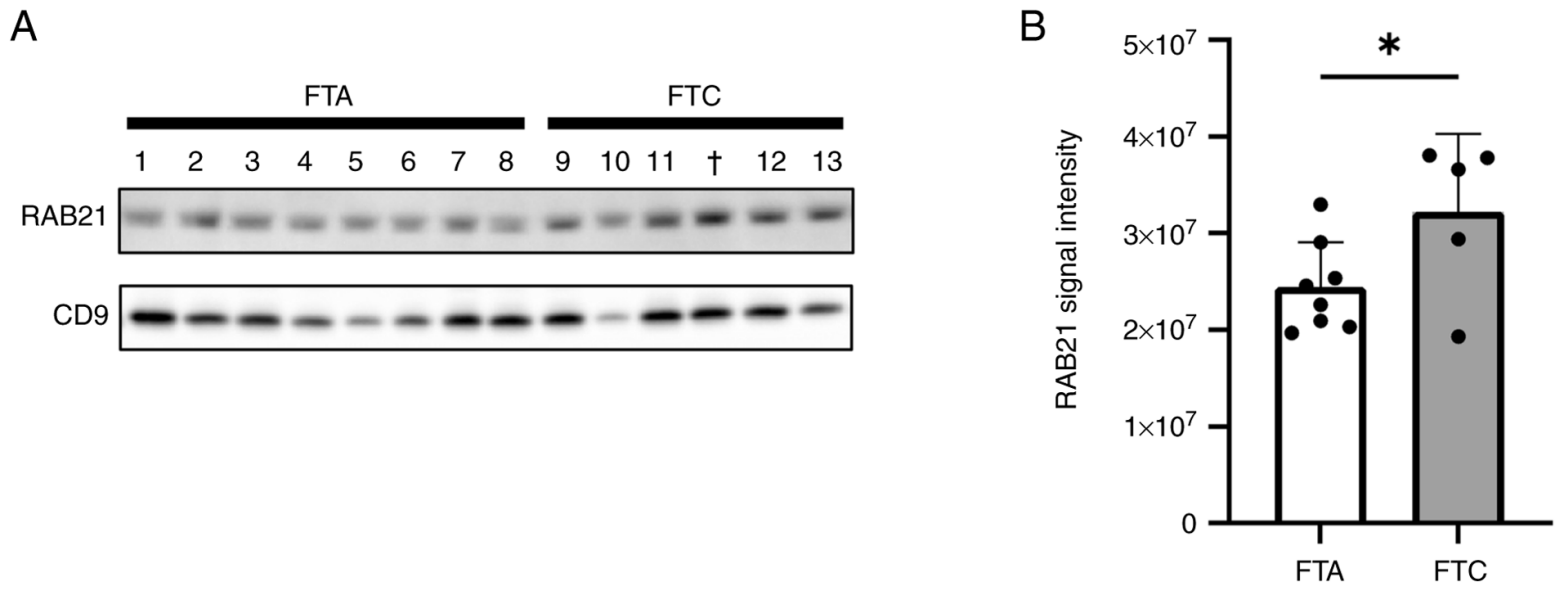
Western blot analysis of RAB21 in serum EVs from patients with FTA and FTC. (A) Representative images of western blotting using anti-RAB21 and anti-CD9 antibodies. Numbers indicate patient samples: FTA (1–8), FTC (9–13); † indicates the FT-UMP case. Uncropped images of blots are shown in Fig. S1B. (B) Bar graph of immunopositive band intensities of RAB21. Data are presented as the mean±SD (FTA, n=8; FTC, n=5). *P<0.05. Densitometry of the band intensities was performed using ImageJ software (version 1.54d). EVs, extracellular vesicles; FTA, follicular thyroid adenoma; FTC, follicular thyroid carcinoma; FT-UMP, follicular tumor of uncertain malignant potential; SD, standard deviation.

RAB21, a member of the Rab family of small GTPases, has been reported to modulate cell adhesion and migration processes (15). Therefore, we focused on RAB21, and attempted to validate its upregulation in FTC serum EVs using western blot analysis, as well as in FTC tissues through *in silico* analyses. First, we examined the RAB21 levels in serum EVs by western blot analysis using CD9 as an EV marker and confirmed that RAB21 was more abundant in the serum EVs of FTC patients than in those of FTA patients (Figs. 3 and S1B). Second, we determined whether RAB21 was actually present in EVs, rather than being a contaminant from the blood, because proteins that are abundant in the blood inevitably contaminate EVs purified from serum. We analyzed the levels of RAB21 in blood using the Protein Atlas database (https://www.proteinatlas.org/humanproteome/blood/proteins+detected+in+ms). Compared with the other upregulated proteins, the RAB21 level was remarkably low (Fig. S2), so we considered that RAB21 was derived from EVs and was not a contaminant from the blood, and that FTC tissues might contain abundant RAB21. Third, we estimated RAB21 mRNA expression in FTC and FTA tissues using the GSE15045 dataset from the GEO public database. This suggests that RAB21 mRNA was significantly upregulated in FTC compared with the level in FTA (Fig. S3). These findings indicate that RAB21 is upregulated in FTC tissues and that its levels are increased in serum EVs from FTC patients.

### Effects of RAB21 knockdown on the malignant phenotype of FTC-133 cells

**Figure 4.**
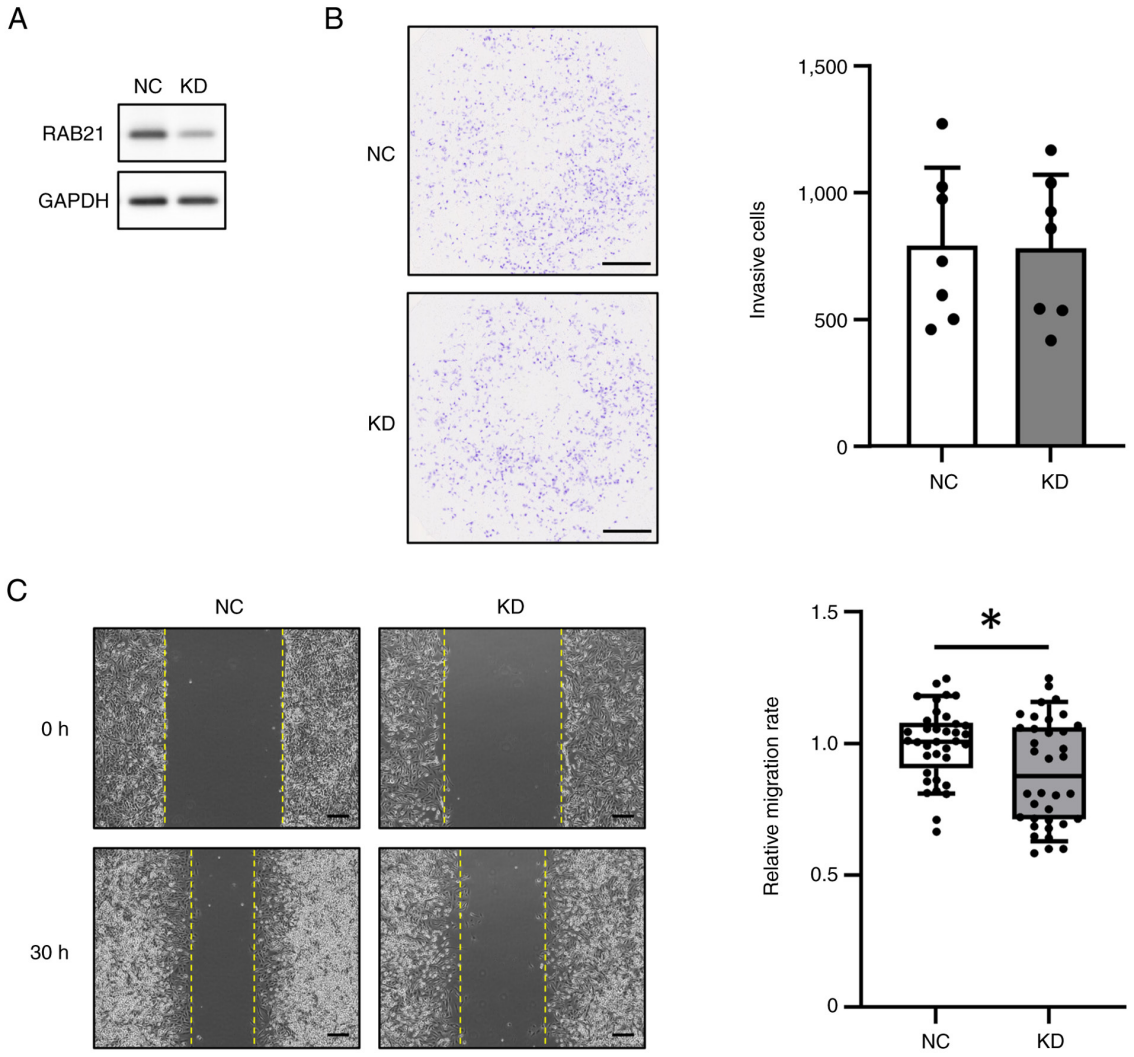
Effect of RAB21 knockdown on FTC-133 cell invasion and migration. (A) Western blot images of RAB21 KD and NC in FTC-133 cells. Uncropped images of blots are shown in Fig. S1C. (B) Cell invasion assay using RAB21-KD cells. The number of cells that passed through the Matrigel layer was determined and compared between RAB21-KD cells and NC cells. Left panels and right bar graph show images of stained cells that passed through the Matrigel layer and a graph of each cell number, respectively. Scale bar, 1 mm. Bar graphs show the mean ± SD (n=7). (C) Cell migration assay of RAB21-KD cells. The cells were cultured for 30 h after being scratched with a pipette tip. The cell migration rate was calculated by comparing the wound areas at 0 and 30 h, as described in the Materials and methods section. Left panels and right box plot show images of cells at 0 and 30 h, and a graph of the cell migration rate compared to the control, respectively. Scale bar, 300 µm. Box plots represent the median, with whiskers extending from the 10th to the 90th percentile (n=36). *P<0.05. KD, knockdown; NC, negative control; SD, standard deviation.

Because RAB21 gene expression was increased in the tissues of FTC patients, we considered that RAB21 might be involved in the malignant phenotype of tumors. Therefore, we examined whether the knockdown (KD) of RAB21 expression in FTC-133 cells, a follicular thyroid carcinoma cell line, might affect malignant phenotypes such as invasion and migration. RAB21-KD in FTC-133 cells was performed using DsiRNA and Lipofectamine RNAiMAX, and confirmed by western blot analysis (Figs. 4A and S1C). Cell invasion was performed using a Matrigel invasion chamber for 24 h. The results showed that the invasion of RAB21-KD cells did not differ from that of the negative control (NC) (Fig. 4B). However, the cell migration rate calculated from the change in wound area during 30 h was significantly decreased in RAB21-KD cells (Fig. 4C). Taken together, these findings suggest that RAB21-KD did not affect cell invasion but reduced the migration of FTC-133 cells under these experimental conditions.

## Discussion

A major problem in the clinical diagnosis of FTC is that discriminating between this malignant carcinoma and benign FTA is difficult. As a result, a definitive diagnosis is typically made only after the surgical removal of the tumor.

Recent efforts to differentiate FTC from FTA have primarily focused on identifying molecular markers using formalin-fixed, paraffin-embedded tissues. Regarding gene markers, genetic mutation analysis revealed FLT3, TP53, and RET were candidate markers for detecting malignancies in follicular lesions (20). Moreover, another study using a gene expression microarray and qRT-PCR identified CPQ, PLVAP, TFF3, and ACVRL1 as potential biomarkers (21). Regarding protein markers, CD56, HBME-1, Galectin-3, and CK19 were reported to be useful for differentiating FTC from FTA based on an immunohistochemical approach (22). Moreover, proteomic analysis demonstrated that ferroptosis pathways were altered in malignant follicular carcinoma (23). Regarding DNA methylation markers, Zhang *et al* (24) identified 70 DNA methylation haplotype block markers, many of which were located in promoter regions (24). Furthermore, combining the detection of thyrotropin receptor mRNA in circulating tumor cells with fine-needle aspiration biopsy results has been reported to improve diagnostic accuracy (25). Additionally, Zabegina *et al* (26) demonstrated that miRNAs of the let-7 family were upregulated in plasma EVs from FTC patients (26).

In this study, we focused on proteins in serum EVs because they might be directly involved in the malignant phenotype of FTC and thus, provide therapeutic targets for FTC. The comparison of protein profiles of serum EVs between FTC and FTA demonstrated that RAB21 protein in serum EVs was elevated in FTC patients and was a potential marker for discriminating FTC preoperatively. Moreover, blood can be collected repeatedly with minimal invasiveness, so this approach might enable the monitoring of disease recurrence (27).

RAB21 is involved in intracellular vesicular trafficking, specifically in the early endosome pathway of endocytosis, the mechanism by which cells internalize external substances (28). In terms of the relationship between RAB21 expression in tissues and cancer, Lin *et al* (29) and Anand *et al* (30) demonstrated that high RAB21 expression was significantly correlated with poor overall survival in breast invasive carcinoma (BRCA) and pancreatic ductal adenocarcinoma (PDAC), using Gene Expression Profiling Interactive Analysis and The Cancer Genome Atlas databases, respectively (29,30). Regarding the intracellular functions of RAB21, Pellinen *et al* (15) reported that it regulated cancer invasion and migration via integrin-β1 in breast cancer cell lines (15). Integrins expressed on the cell surface provide adhesion between cells and the extracellular matrix (31). Mai *et al* (32) revealed that RAB21 indirectly influenced cancer cell invasion and migration capabilities by mediating integrin endocytosis in breast cancer cells, forming part of a dynamic trafficking system where its competitive binding to p120RasGAP determined integrin localization between endosomes and the cell surface (32). While RAB21 knockdown affected cell migration in our study, no significant difference was observed in invasion. This observation suggests that RAB21 primarily regulates integrin-mediated migration, whereas invasion requires additional processes such as extracellular matrix degradation through matrix metalloproteinases (33). In addition, the silencing of RAB21 in glioma cell lines has been shown to induce apoptosis and inhibit cell proliferation (34). Pei *et al* (35) also demonstrated that RAB21 plays a crucial role in regulating the recycling of glucose transporter SLC2A1/GLUT1 to the cell membrane, thus maintaining glucose uptake and energy homeostasis in cancer cells. Their findings suggested that RAB21 is essential for cancer cell survival and proliferation, particularly in the glucose-deprived tumor microenvironment (35). Moreover, a recent study reported that EVs derived from head and neck squamous cell carcinoma carrying RAB21 homed to lung macrophages and were incorporated into them through an interaction with integrin-β1 on the macrophage surface, eventually inducing the immunosuppression of these cells (36). These findings suggest that increased RAB21 expression may contribute to the malignant progression of tumors. Therefore, we analyzed the function of RAB21 in malignant FTC-133 cells and found that RAB21 knockdown reduced their migratory ability. The results are consistent with those of previous studies, and indicate that RAB21 may be involved in the malignant phenotype of FTC.

Our study had several limitations. The Tim4-based phosphatidylserine affinity method may introduce false-positive results because of potential contamination and false-negative findings by missing certain EV subpopulations. Sample collection limited to specific Japanese hospitals may create geographic and demographic biases, potentially restricting the generalizability of the results. Additionally, our relatively small sample size affects statistical power and reliability, and certain experimental conditions may require further optimization. Future research should employ multiple EV isolation techniques in larger, more diverse cohorts through multi-center collaborations to validate and extend our findings.

In conclusion, this study searched for biomarkers to distinguish between FTC and FTA, focusing on the proteins contained in serum EVs. Based on the proteomic analysis of serum EVs in patients and functional cell culture studies, we found that RAB21 in serum EVs might be a discriminant marker for FTC and that it could play an important role in the malignant phenotype of FTC. Further larger-scale clinical studies are necessary to validate our findings; however, to the best of our knowledge, this is the first study to describe a protein marker found in serum EVs with the potential to discriminate between FTC and FTA. This is particularly valuable because serum EVs are readily available for preoperative evaluation via a minimally invasive procedure. Moreover, our findings on the functional role of RAB21 in cancer cell migration provide insights into the molecular mechanisms underlying the malignant progression of FTC, potentially opening new avenues for therapeutic interventions.

## Supplementary Material

Supporting Data

Supporting Data

## Figures and Tables

**Table I.** Up- and downregulated proteins in serum EVs of FTC patients isolated using PS affinity method.

Description	Gene symbol	log_2_ (FTC/FTA)	P-value of FTC/FTA
Keratin, type I cytoskeletal 13	KRT13	3.79	0.032
Lipopolysaccharide-binding protein	LBP	2.10	0.011
Serum amyloid A-1 protein	SAA1	1.99	0.029
N-acetylmuramoyl-L-alanine amidase	PGLYRP2	1.63	0.031
Ras-related protein Rab-21	RAB21	1.60	0.015
Fibrinogen alpha chain	FGA	1.48	0.014
Transaldolase	TALDO1	−1.01	0.015
Peroxiredoxin-5, mitochondrial	PRDX5	−1.02	0.036
Isoform 1 of Four and a half LIM domains protein 1	FHL1	−1.19	0.046
Kinesin-like protein KIF2A	KIF2A	−1.21	0.019
Rho GDP-dissociation inhibitor 1	ARHGDIA	−1.30	0.033
Beta-actin-like protein 2	ACTBL2	−1.45	0.038
Coronin-1B	CORO1B	−1.66	0.034
SH3 domain-binding glutamic acid-rich-like protein 2	SH3BGRL2	−1.93	0.016
Immunoglobulin heavy variable 1–8	IGHV1-8	−2.35	0.036
Crk-like protein	CRKL	−2.39	0.002
Superoxide dismutase [Cu-Zn]	SOD1	−2.44	0.006
Ficolin-2	FCN2	−6.21	0.009

## Data Availability

The data generated in the present study may be requested from the corresponding author. The mass spectrometry proteomics data generated in the present study may be found in the ProteomeXchange Consortium via the jPOST partner repository under accession numbers PXD064847 for ProteomeXchange and JPST003852 for jPOST or at the following URL: https://repository.jpostdb.org/entry/JPST003852.

## Abbreviations

AN: adenomatous nodule
DsiRNAs: Dicer-substrate short interfering RNAs
EVs: extracellular vesicles
FA: formic acid
FBS: fetal bovine serum
FTA: follicular thyroid adenoma
FTC: follicular thyroid carcinoma
FT-UMP: follicular tumor of uncertain malignant potential
GEO: Gene Expression Omnibus
HRP: horseradish peroxidase
KD: knockdown
nanoLC-MS/MS: nano liquid chromatography-tandem mass spectrometry
PBS: phosphate-buffered saline
PTC: papillary thyroid carcinoma
RT: room temperature
TBS: Tris-buffered saline
TEAB: triethylammonium bicarbonate
