# Supplementary material for: Proteomics analysis reveals elevated RAB21 in serum-derived extracellular vesicles from patients with follicular thyroid carcinoma
Source: Oncol Lett. 2025 Jul 16;30(3):444. doi: 10.3892/ol.2025.15190 (PMC12308455; doi:10.3892/ol.2025.15190)
Supplement: Supporting Data [file Supplementary_Data1.pdf]

Figure S1. Uncropped western blot data. Uncropped images of western blot displayed in Fig. 1A (A), Fig. 3A (B), and Fig. 4A (C) are shown. The areas enclosed by red dashed lines indicate the portions used in the figures.

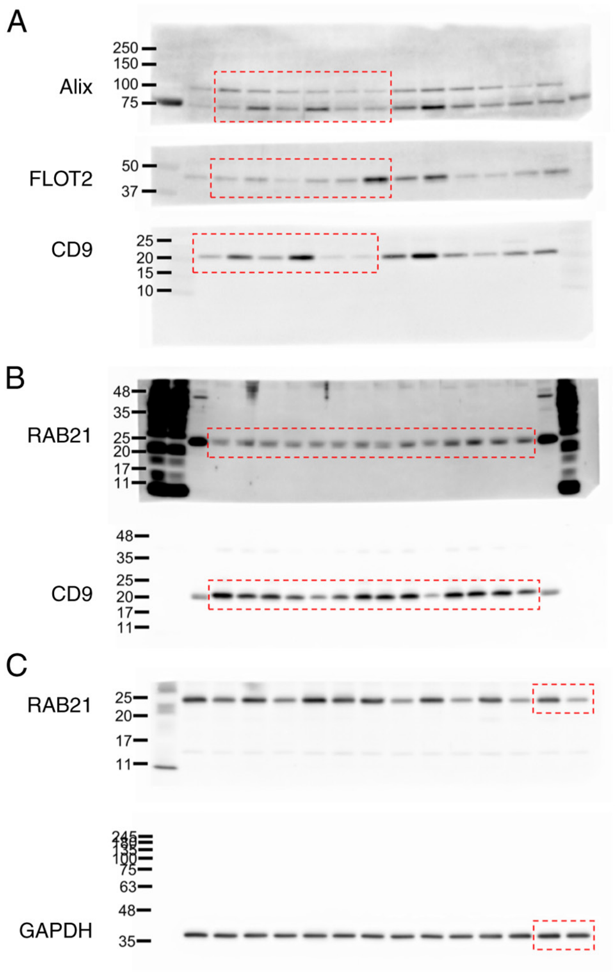

Figure S2. Plasma concentrations of proteins extracted from the Protein Atlas database. Proteins that were significantly abundant in serum EVs from FTC patients were subjected to analysis. The left table summarizes the plasma concentrations of the focused proteins and their ranks among 4,066 registered proteins. The right chart displays the distribution of all 4,066 proteins registered in the database. The positions of the proteins of interest are highlighted within the overall distribution. The URL for plasma concentration of proteins in the Protein Atlas database is as follows: https://www.proteinatlas.org/humanproteome/blood/proteins+detected+in+ms. EVs, extracellular vesicles; FTC, follicular thyroid carcinoma.

Gene symbol	Concentration	Rank from top among 4,066 proteins
KRT13	680 µg/l	319/4,066
LBP	1.3 mg/l	267/4,066
SAA1	11 mg/l	109/4,066
PGLYRP2	87 mg/l	30/4,066
RAB21	13 ng/l	3,858/4,066
FGA	140 mg/l	10/4,066

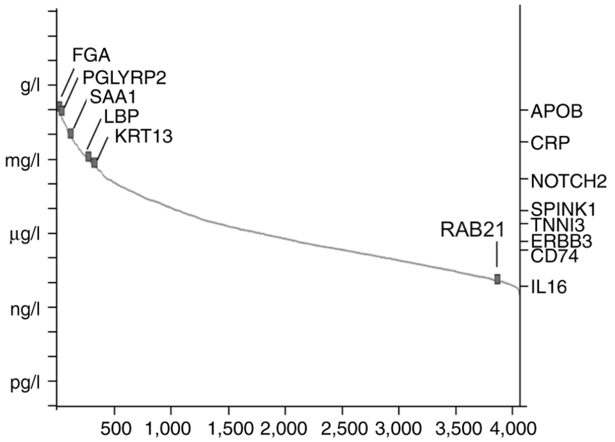

Figure S3. RAB21 mRNA expression extracted from the GEO public database. The mRNA expression values of RAB21 in FTA and FTC tissues were analyzed using the GSE15045 dataset from the GEO public database. Analysis was performed using the GEO2R website (https://www.ncbi.nlm.nih.gov/geo/geo2r/). The left bar graph shows the mRNA expression values of RAB21, and the right table summarizes the demographic characteristics and clinical backgrounds of each subject from the database. Gene expression data were analyzed using Welch's t-test. *P<0.05. FTA, follicular thyroid adenoma; FTC, follicular thyroid carcinoma; GEO, Gene Expression Omnibus.

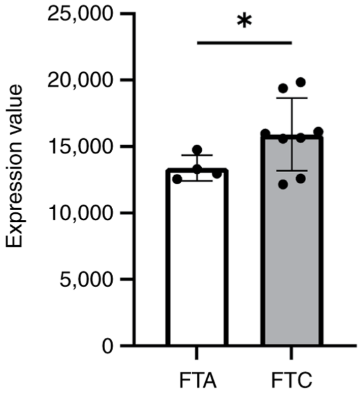

GEO_accession	Sex	Age	Pathology	Group
GSM375747	F	34	follicular thyroid adenoma	FTA
GSM375748	F	44	follicular thyroid adenoma	FTA
GSM375749	F	39	follicular thyroid adenoma	FTA
GSM375750	F	39	follicular thyroid adenoma	FTA
GSM375751	F	34	invasive follicular thyroid carcinoma	FTC
GSM375752	M	60	invasive follicular thyroid carcinoma	FTC
GSM375753	F	17	invasive follicular thyroid carcinoma	FTC
GSM375754	F	51	invasive follicular thyroid carcinoma	FTC
GSM375755	M	54	minimal invasive follicular thyroid carcinoma	FTC
GSM375756	F	47	minimal invasive follicular thyroid carcinoma	FTC
GSM375757	F	56	minimal invasive follicular thyroid carcinoma	FTC
GSM375758	M	39	minimal invasive follicular thyroid carcinoma	FTC
